# Modular cone-and-plate device for mechanofluidic assays in Transwell inserts

**DOI:** 10.3389/fbioe.2025.1494553

**Published:** 2025-01-27

**Authors:** Daniel Chavarria, Kissamy A. Georges, Brian J. O’Grady, Khalid K. Hassan, Ethan S. Lippmann

**Affiliations:** ^1^ Department of Chemical and Biomolecular Engineering, Vanderbilt University, Nashville, TN, United States; ^2^ Department of Bioengineering, University of Massachusetts Dartmouth, Dartmouth, MA, United States; ^3^ School for Science and Math at Vanderbilt, Vanderbilt University, Nashville, TN, United States; ^4^ Department of Biomedical Engineering, Vanderbilt University, Nashville, TN, United States; ^5^ Vanderbilt Brain Institute, Vanderbilt University, Nashville, TN, United States; ^6^ Department of Neurology, Vanderbilt University Medical Center, Nashville, TN, United States; ^7^ Interdisciplinary Materials Science Program, Vanderbilt University, Nashville, TN, United States; ^8^ Vanderbilt Memory and Alzheimer’s Center, Vanderbilt University Medical Center, Nashville, TN, United States

**Keywords:** blood-brain barrier, shear stress, Transwell^®^, endothelial cell, mechanobiology, blood flow, cone-and-plate

## Abstract

In this work, we present a cost effective and open-source modular cone-and-plate (MoCAP) device that incorporates shear stress in the popular Transwell^®^ insert system. This system acts as a lid that incorporates flow into 24-well Transwell^®^ inserts while preserving the ability to conduct molecular profiling assays. Moreover, the MoCAP device can be rapidly reconfigured to test multiple shear stress profiles within a single device. To demonstrate the utility of the MoCAP, we conducted select assays on several different brain microvascular endothelial cell (BMEC) lines that comprise models of the blood-brain barrier (BBB), since shear stress can play an important role in BBB function. Our results characterize how shear stress modulates passive barrier function and GLUT1 expression across the different BMEC lines. Overall, we anticipate this low cost mechanofluidic device will be useful to the mechanobiology community.

## 1 Introduction

*In vitro* models that require assessments of barrier function primarily rely on Transwell^®^ inserts, which are widely used due to their versatility and ease of implementation (Stone et al., 2019; Petrovskaya et al., 2022; Nakayama-Kitamura et al., 2023; Kim W. et al., 2022; Mosiagina et al., 2023; Kikuchi et al., 2019; Park et al., 2023; You et al., 2022; Haileselassie et al., 2020; Li et al., 2023; Han et al., 2024). Transwell^®^ inserts allow investigators to conduct a variety of assays related to passive and active barrier function, including transendothelial electrical resistance (TEER) and permeability measurements. Cells on Transwell^®^ filters are also readily accessible for molecular assays, including immunofluorescence staining, Western blots, and quantitative polymerase chain-reaction (qPCR). A major disadvantage of Transwell^®^ inserts is that cells are cultured under static conditions, lacking shear stress to mimic the hemodynamic effects of fluid flow, although some prior efforts have sought to overcome this issue. For example, Kim and colleagues utilized an annular shaker device to house Transwell^®^ inserts and introduce fluid flow over immortalized brain microvascular endothelial cells (BMECs) as a blood-brain barrier (BBB) model, which resulted in higher mRNA levels of tight junctions, a subtle but significant increase in TEER measurements, and decreased paracellular permeability of lucifer yellow (Kim et al., 2024). As another example, Bolden and colleagues incorporated shear stress into Transwell^®^ inserts using a custom fluidic device that could house Transwell^®^ inserts. Using this device, they mimicked reperfusion analogous to a traumatic brain injury and observed a significant decrease in TEER measurements in a triculture of primary BMECs, bone marrow-derived mesenchymal stem cells, and astrocytes (Bolden et al., 2023). As an alternate to Transwell^®^ setups, microfluidic setups have been used to incorporate shear stress to better recapitulate physiology (Hajal et al., 2022; Lauranzano et al., 2022; Yang et al., 2023; Shi et al., 2023; Straehla et al., 2022; Kadry et al., 2024; Hudecz et al., 2023; Yang et al., 2024; Fan et al., 2023; Meena et al., 2022; Westerhof et al., 2023; Koo et al., 2018; Campisi et al., 2018; Tang et al., 2020; Lee et al., 2020; Booth and Kim, 2012). However, most microfluidic models substitute throughput capacity for the ability to better recapitulate physiological shear stress conditions. Additionally, current microfluidic models, as well as the Transwell^®^ models detailed above, are limited to testing a singular shear stress condition per device. This hampers the number of shear stress conditions that can be tested at a time. Thus, an unmet need for the field is a mechanofluidic device that facilitates testing of multiple shear stress conditions simultaneously. Ideally, this device would also be compatible with commercially available Transwell^®^ inserts to facilitate adoption by the broader research community.

Here, we present a modular cone-and-plate (MoCAP) mechanofluidic device that is compatible with Transwell^®^ inserts and can test multiple shear stress conditions within a 24-well plate setup. We showcase the ease of use of this MoCAP device and its compatibility with the current workflow of Transwell^®^ inserts by evaluating *in vitro* models of the BBB. The BBB is a restrictive physiological interface between the brain vasculature and parenchyma that plays a crucial role in brain homeostasis (Abbott and Friedman, 2012). The BBB is composed of multiple cell types including BMECs, which are the primary barrier-forming unit of the BBB. Blood flow generates a tangential force that runs parallel to the lumen of BMECs, directly exerting this biophysical shear stress on the vascular endothelium. The hemodynamic effects of blood flow are known to influence the properties of the BBB, where shear stress has been shown to regulate BMEC cell adhesion, tight junction expression, and transporter expression and activity, which influence drug permeability (Cucullo et al., 2011; Santa-Maria et al., 2021; Choublier et al., 2022; Luissint et al., 2012). As such, we used the MoCAP to evaluate how different continuous shear stress (CSS) and pulsatile shear stress (PSS) profiles influence select BBB properties across various BMEC lines. Overall, because the MoCAP device is cost efficient and user friendly, we anticipate it will be a useful mechanobiological tool for studying shear effects at biological interfaces, including the BBB.

## 2 Materials and methods

### 2.1 Fabrication of MoCAP device

All components for the MoCAP device were designed using a computer aided design software (Autodesk Inc., One Market, Suite 400, San Francisco, CA 94105). A bill of materials lists all the necessary hardware needed to build a fully functional MoCAP (Table 1). Components of MoCAP device were printed using a stereolithography Form 3+ 3D printer (Formlabs Inc., 35 Medford Street, Suite 201, Somerville, MA 02143) and high-temperature resin (RS-F2-HTAM-02). Low angle cones were coated with parylene-C using a parylene deposition machine (Specialty Coating Systems, 7,645 Woodland Drive, Indianapolis, IN 46278) to ensure biocompatibility (O’Grady et al., 2021). Commercially available bearings (McMaster-Carr Supply Co., 2,828 No Paulina St, Chicago, IL 60657) were purchased and utilized in the device. The gearbox within the device was lubricated utilizing a small amount of medical grade petroleum jelly (Covidiein, 15 Hampshire Street, Mansfield, MA 02048). The gearbox in the MoCAP device was driven by a NEMA 17 stepper motor (STEPPERONLINE Inc., 228 Park Ave S 79525, New York, NY 10003, United States) and held with four stainless steel screws (McMaster-Carr Supply Co., 2,828 No Paulina St, Chicago, IL 60657). The software for the MoCAP device was developed in-house utilizing a programming language (Python Software Foundation, 512 Lafayette Boulevard, Suite 2, Fredericksburg, Virginia 2,240). The software was based on a prior design used to control a spinning bioreactor for brain organoid culture (Romero-Morales et al., 2019).

**TABLE 1:** Bill of materials.

Part name	Link	Cost	Quantity
High Temperature Resin	https://formlabs.com/store/materials/high-temp-resin/	$199.00	1
Bearings	https://www.mcmaster.com/7804K113/	$6.46	48
Nema17 Motor	Amazon:Nema17 Motor	$9.62	1
Easy Driver for Motor	https://www.amazon.com/SparkFun-EasyDriver-Stepper-Motor-Driver/dp/B004G4XR60#customerReviews	$22.92	3
Raspberry Pi 3 A+	Raspberry Pi 3 Amps+| McMaster-Carr	$31.25	1
Breakout Board	Amazon.com: GPIO Breakout Board HAT for Raspberry Pi	$18.99	1
2 Wire Connectors (2.45 mm)	amazon.com: 2 wire Female-Connections-JST	$9.99	1
3 Wire Connectors (2.45 mm)	amazon.com: 3 wire Female-Connections-JST	$8.59	1
4 Wire Connectors (2.45 mm)	amazon.com: 4 wire Female-Connections-JST	$8.59	1
Raspberry Pi Touch Test	Amazon.com: Raspberry-Pi-7-Touchtest-Display	$75.81	1
Solder Seal Wire Connectors	Amazon.com: Solder Seal Connectors	$31.99	1
Fans (30 Mm 5v)	Amazon.com: Cooling 5V Fans	$19.99	1
Hex Standoff	mcmaster.com: Hex standoff	$5.18	2
316 Stainless Steel Socket Head Screw	mcmaster.com: Head Screw	$15.60	1
Female To Female 2.54 mm Jumper Wires	Amazon.com: Fem to Fem jump wires	$11.98	1
12v/24v to 5v Power Converter Dc-Dc With Micro-USB	Amazon.com: 12V to 5V power converter	$9.99	1
Toggle Button for On/Off Power	Amazon.com: on off toggle switch	$7.99	1
Washers For 3 mm Screw	https://www.mcmaster.com/98689A112/	$3.42	1
M3 X 12 mm Flat Head Socket Screws	Amazon.com: M3 Head Screw	$6.55	1
Power Cord	Amazon.com: 12V Power Cord	$15.99	1
Inline Switch	Amazon.com: Inline Switch	$8.99	1
4 Pin JST Male to Female Wire Connectors	Amazon.com: JST 4 pin connectors	$12.99	1
SD Card Pre-Loaded with Pi Operating System	Amazon.com: Raspberry Pi Preloaded SD Card	$9.99	1
Power Jack Cord Socket	Amazon.com: Power Jack Cord Socket	$8.99	1
4 Pin Male to Female JST Plugs	Amazon.com: 4 pin JST Plugs M to F	$9.99	1
Total Cost as of 08/07/2024		$ 925.49	

### 2.2 Cell culture

All cells were grown in a standard humidified incubator (5% CO_2_, 37°C). Primary BMECs (ACBRI 376) and immortalized BMECs (hCMEC/D3) (Weksler et al., 2005) were cultured and expanded with endothelial cell growth medium (R&D Systems) supplemented with Endothelial Cell Growth Supplement (R&D Systems), 10% fetal bovine serum (Thermo Fisher), GlutaMAX (Thermo Fisher), and gentamycin (25 μg/mL; Thermo Fisher). Primary and immortalized BMECs were seeded at 100,000 cells/cm^2^ onto polyester Transwell^®^ filters (3,470; 0.4 µm pore size; Corning) coated with collagen IV (400 μg/mL; Sigma-Aldrich) and fibronectin (100 μg/mL; Sigma-Aldrich). Twenty-four hours later, the medium was changed to remove floating cells. Induced pluripotent stem cells (iPSCs; CC3 line) were cultured and seeded as previously published (Neal et al., 2019; Hollmann et al., 2017). Briefly, iPSCs were seeded onto Matrigel-coated plates with E8 medium supplemented with Y-27632 (Tocris) at a cell density of 15,600 cells/cm^2^. Cells were differentiated 24 h after seeding by changing to E6 medium. E6 medium was replenished daily for 4 days. Then, cells were switched to human endothelial serum-free medium (hESFM; Thermo Fisher Scientific) supplemented with 10 µM retinoic acid (RA; Sigma-Aldrich), 20 ng/mL human basic fibroblast growth factor (bFGF; Peprotech), and B27 supplement (Thermo Fisher Scientific). Medium was not changed for 48 h. Then, cells were collected and seeded at 100,000 cells/cm^2^ onto polyester Transwell^®^ filters (3,470; 0.4 µm pore size; Corning) coated with collagen IV (400 μg/mL; Sigma-Aldrich) and fibronectin (100 μg/mL; Sigma-Aldrich). Twenty-four hours after seeding, RA and bFGF were removed from the medium to induce barrier phenotype. For all experiments, media volumes were 200 μL and 600 µL in the apical and basolateral chambers of the Transwell^®^ insert, respectively.

### 2.3 Increasing media viscosity using dextran

To increase the cell media viscosity to 3 mPa (6.5% dextran w/w), we referenced data from two publications (Rouleau et al., 2010; Li et al., 2008), created a concentration curve with the data, and performed a non-linear regression analysis to calculate the concentration that would yield our desired media viscosity (Supplementary Figure S1). Dextran powder (40 kDa; Sigma-Aldrich) was dissolved in non-supplemented media utilizing a heat plate (40°C) and sterilized by vacuum filtration using a pre-heated filter (Sigma-Aldrich).

### 2.4 Mechanofluidic assay using MoCAP device

The MoCAP device was sterilized in an autoclave before each experiment. The motor is removed from the hardware and sterilized separately with 70% ethanol. Cells were cultured on 24-well Transwell^®^ inserts for 2 days, as detailed above, before exposure to flow. Cells were then continuously exposed to a defined CSS or PSS profile except for the brief daily period when TEER measurements were acquired. Culture medium (50 µL) was added to the apical chamber of filters each day to account for media loss during TEER measurements.

### 2.5 Calculating shear stress utilizing analytical evaluations

To calculate the shear stress induced by the MoCAP device, we utilized the analytical solution for a cone-and-plate system as previously described in the literature (Franzoni et al., 2016; Sucosky et al., 2008). The following formula was used to estimate shear stress and calibrate the angular of the velocity of the MoCAP device:

$$\tau =µ\;\frac{\omega \;r}{h+r\;\tan\alpha \;}$$

Here, τ is shear stress, µ is the dynamic viscosity of the cell media (3 mPa), ω is angular velocity, h is the gap between the tip of the low angle cone and the cells (200 µm), r is the cone radius (0.29 cm), and α is the angle of the cone (2°). The angular velocity was adjusted to account for the dynamic viscosity of the cell media utilized during experimentation. Supplementary Figure S2A shows these parameters in the context of a single cone within a Transwell^®^ insert. While the constant distance between the cone and cells is assumed, it is understood that Transwell^®^ inserts are not perfectly flat and can move slightly within the cell culture well; thus, the shear stress is an estimate based on the constant distance assumption.

### 2.6 Calibration of MoCAP device

To calibrate the MoCAP device, an initial set of low angle cones with an arbitrary shaft length were printed and parylene coated. Plates were seeded with BMECs, and the MoCAP was placed on top of the plates and run overnight. The next day, the MoCAP device was removed, and the plates were inspected for cell monolayer damage under a phase contrast microscope. The experiment was then repeated with sequential additions of 100-micron shims (McMaster-Carr Supply Co.; 90214A111) until no scratches were detected in the BMEC monolayers. The low angle cones were then reprinted taking into the account the number of shims utilized to achieve zero damage to the cell monolayer. This final gap of 200 µm between the tip of the low angle cone and cells was utilized for all experimentation. Once fully assembled, reflective tape was placed on top of the shaft cap to verify that the input angular velocity of the software matched the output angular velocity of the motor utilizing a digital tachometer (NEIKO Tools, Taiwan).

### 2.7 Calculating shear stress delay due to gear backlash

To calculate the shear stress delay caused by gear backlash within the MoCAP device, we utilized the following formula:

$$Lag\;Time=\frac{∆\theta \;\left(backlash\right)}{\omega_{input}}$$

Here, ∆θ represents the gear backlash angle and $\omega_{input}$ represents the angular velocity input in the MoCAP device. The gear backlash angle was measured to be 2.2° in the 3D printed gears utilized in the MoCAP device.

### 2.8 TEER measurements

TEER measurements were taken daily during shear stress treatments. Prior to measurements, the MoCAP was removed from the filters and cells were equilibrated at room temperature for 10 min. Then, measurements were acquired with a commercially available electrode system (World Precision Instruments) with a chopstick configuration (STX2). Chopsticks were carefully inserted into the Transwell^®^ filters to avoid scratching the cell monolayer. The measurements were recorded after the signal had stabilized. The reported TEER (T_E_) was determined with the following formula:

$$T_{E}=\left(T_{M}-T_{B}\right)\times Area$$

The measured TEER from an endothelial monolayer (T_M_) was subtracted by the measured TEER from a blank Transwell^®^ insert with no cells (T_B_). This quantity was then multiplied by the surface area of the Transwell^®^ insert (0.33 cm^2^) to determine T_E_. All TEER measurements in this study are reported as Ω × cm^2^.

### 2.9 Immunofluorescent staining

Following the mechanofluidic assays, cells were immediately fixed with 4% paraformaldehyde for 5 min. Cells were then washed three times with phosphate buffered saline (PBS). Once rinsed, cells were permeabilized with PBS containing 0.3% Triton X-100 for 5 min. After permeabilizing, the cells were blocked with PBS containing 10% goat serum for 60 min. Cells were then incubated with fluorescent-conjugated anti-GLUT1 antibody or phalloidin in PBS containing 10% goat serum overnight at 4°C on a shaker (R&D Systems FAB1418G, 1:250; Thermo Fisher Scientific A12379, 1:250). The next day, cells were rinsed three times with PBS and incubated with DAPI to label nuclei (Thermo Fisher Scientific 62248, 1:1,000), then rinsed three final times with PBS. The Transwell^®^ inserts were then transferred into a 12-well glass bottom plate (Cellvis). To improve imaging quality, a solution of 2.5 M fructose and 60% glycerol was used as the final imaging medium on the apical and basolateral sides of the Transwell^®^ inserts (Dekkers et al., 2019). Cells were imaged utilizing a Leica DMi8 epifluorescent microscope.

### 2.10 Statistical analysis

All experimental results are shown as mean ± standard error of the mean (SEM). Multiple comparisons between groups were analyzed by two-way ANOVA followed by a Bonferroni’s *post hoc* test. A two-tailed probability value p < 0.05 was considered statistically significant. An independent replicate for each experiment was considered as a singular Transwell^®^ insert. One MoCAP run refers to an experiment utilizing one MoCAP device applying shear stress across a plate of 24-well Transwell^®^ inserts.

## 3 Results

### 3.1 Designing and testing the MoCAP device

**FIGURE 1:**
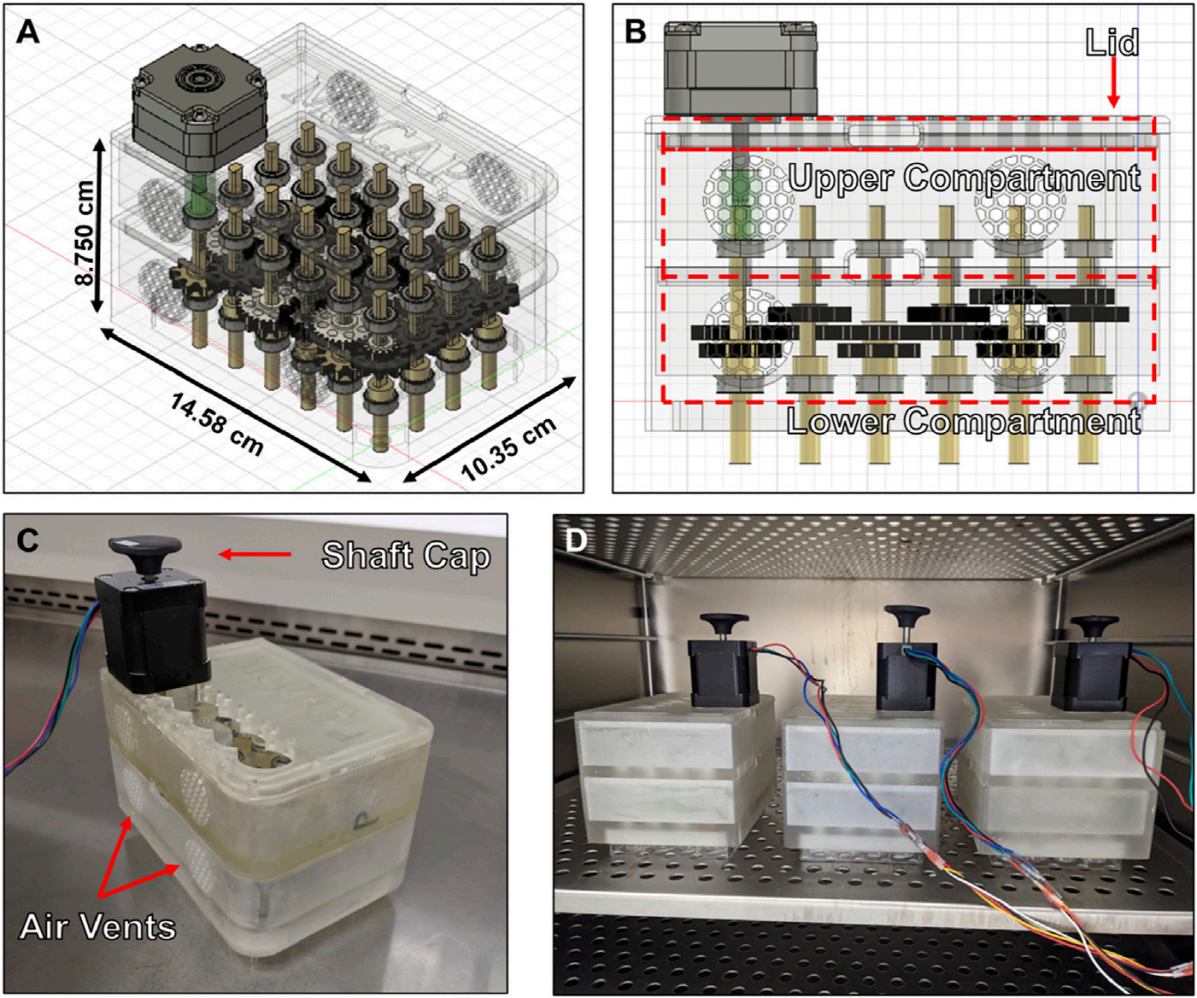
Diagram and pictures of Modular Cone-and-Plate (MoCAP) mechanofluidic device. **(A, B)** Diagrams generated utilizing a computer-aided design program showing a translucent angled **(A)** and side view **(B)** of the MoCAP mechanofluidic device. **(C)** Picture of 3D printed MoCAP device prototype fully assembled. **(D)** Picture of three fully assembled and operational MoCAP devices inside of a cell culture incubator operating of one set of electronics.

We created a modular cone-and-plate (MoCAP) mechanofluidic device that is compatible with the 24-well Transwell^®^ insert system (Figure 1A). The MoCAP device consists of a nesting three-part housing body made up of a lid, an upper housing, and a lower housing (Figure 1B). A NEMA 17 stepper motor can be mounted to the lid in multiple column positions to allow for rapid reposition using M3 stainless steel screws. A key and lock geometry were utilized on custom made gears with a variety of gear ratios (1:1 and 2:1), as well as on the shaft of the low angle cones that were 3D printed, to allow users to quickly interchange components without the need for screws. A 2° low angle was utilized on the cones, as this design has been extensively characterized in the literature, (Chavarria et al., 2023; Spruell and Baker, 2013; Spencer et al., 2016) and the low angle cones were parylene coated to ensure biocompatibility (O’Grady et al., 2021). Bearings were press fitted into designed recesses on the lower and upper housings units to reduce friction during the rotation of the cones. All components of the MoCAP device (except the bearings and NEMA17 motor) were 3D printed with high-temperature resin, which allows for the device to be sterilized with an autoclave. Once fully assembled, the MoCAP device can be placed directly on top of a 24-well Transwell^®^ insert system, acting as a plate lid (Figure 1C). A final gap of 200 µm between the tip of the low angle cone and the cells was utilized to prevent any accidental scratching of the cell monolayer when operating and removing the MoCAP device from the Transwell^®^ inserts. The analytical evaluation of this gap configuration, taking into account the thicknesses of endothelial cells that are reported to range from 0.1–10 µm (Félétou, 2011), demonstrated a negligible difference in the shear stress produced by the MoCAP device at the cell surface creating similar shear stress profiles with a maximum shear stress value closer to the edges of the Transwell^®^ inserts and a dead point at the center of the Transwell^®^ insert (Supplementary Figure S2B). A shaft cap was printed and placed on top of the double shaft NEMA17 motor to visualize the rotation of the motor shaft (Figure 1C). Reflective tape was placed on top of the shaft cap to verify the input angular velocity of the software matched the output angular velocity of the motor utilizing a digital tachometer. The electronics and software were made in-house based on prior designs (Romero-Morales et al., 2019; O’Grady et al., 2018) and allow for the simultaneous operation of up to three MoCAP devices (Figure 1D).

**FIGURE 2:**
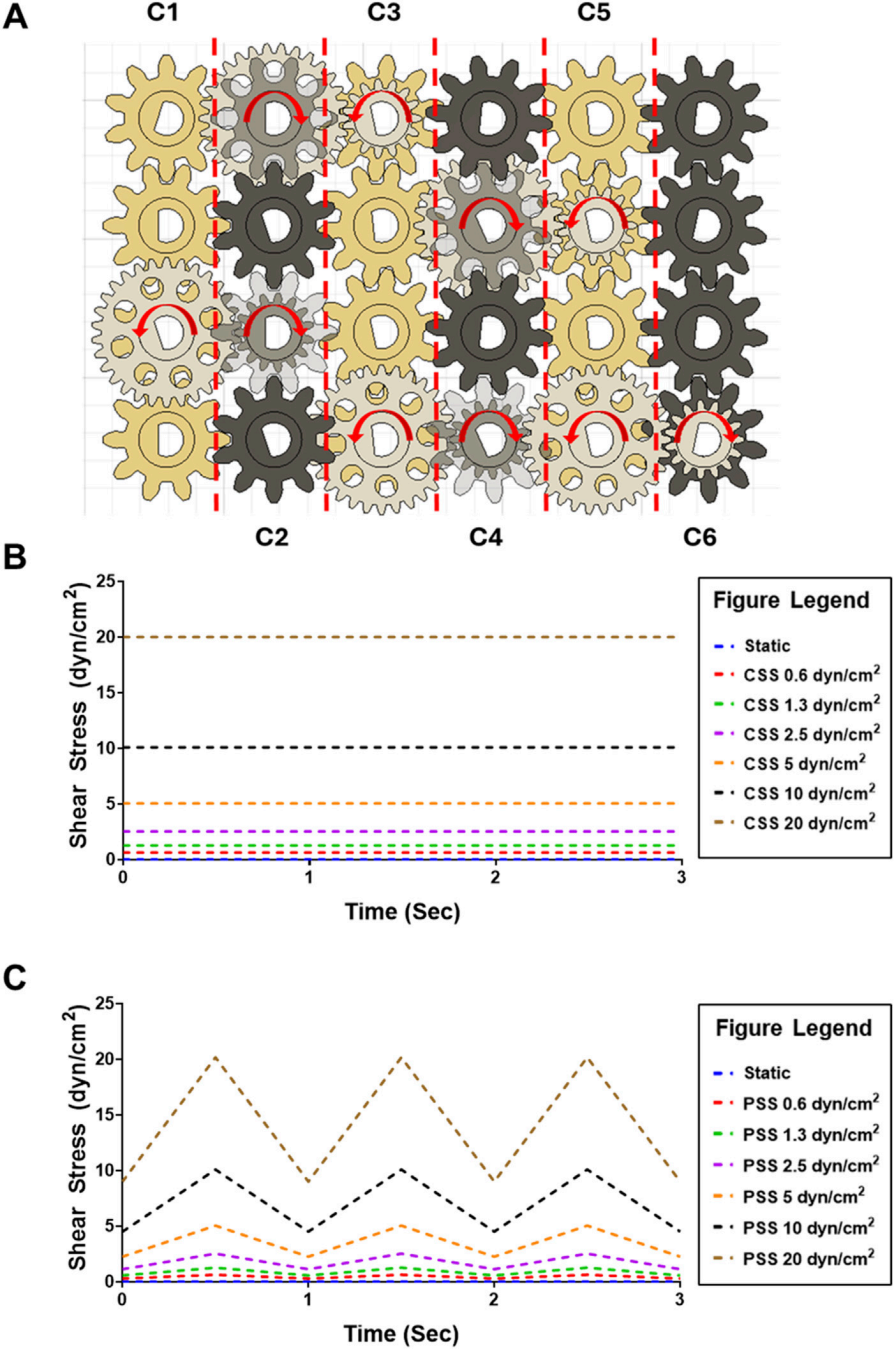
Diagram of MoCAP device gearbox for the generation of multiple shear stress magnitudes and flow profiles. **(A)** Diagram of the top-view of the MoCAP gearbox illustrating the different gear ratio combinations. The white and black gears are offset on height allowing for their rotation without interference. By modifying the gear ratio between the gears connecting adjacent columns (shown in blue), the MoCAP device can double the initial rotational velocity every column. This rotational velocity of the low angle cones is directly proportional to the shear stress generated at the cell surface. **(B)** Plot of predicted maximum continuous shear stress profiles that can be generated by the MoCAP device inside a Transwell^®^ insert. **(C)** Plot of predicted maximum pulsatile shear stress profiles that can be generated by the MoCAP device inside a Transwell^®^ insert (1 Hz frequency).

The MoCAP device generates different shear stress magnitudes at each column by changing the gear ratio between the gears connecting adjacent columns. For example, the gears illustrated in gold in column C1 consist of a 1:1 gear ratio, resulting in the transfer of the same angular velocity from one gear to its neighboring connecting gear down the column (Figure 2A). The white gears illustrated on the diagram are staggered on height placement, allowing the gold and white gears to spin in different planes without interference (Figure 1B). The white gears connecting adjacent columns on the diagram have a 2:1 gear ratio, therefore multiplying the angular velocity from left to the right column by a factor of two. On the diagram shown, we start with an input of 28 rotations per minute (RPM), corresponding to 0.6 dyn/cm^2^ from the NEMA17 motor that is doubled every column, which yields 32 times the initial angular velocity (896 RPM) at the last column in the MoCAP gearbox system (Figure 2A). This gear ratio principle allows the MoCAP device to achieve angular velocities of 28, 56, 112, 224, 448, and 896 RPM, with the corresponding shear stress values of 0.6, 1.3, 2.5, 5, 10, and 20 dyn/cm^2^ at columns C1, C2, C3, C4, C5, and C6, respectively (Table 2). Thus, when the MoCAP device is run at a constant angular velocity, we can generate CSS ranging from 0.6 to 20 dyn/cm^2^ by filling the entire gearbox with these 1:2 gear ratios between columns (Figure 2B). The maximum shear stress the MoCAP can generate using this configuration is 20 dyn/cm^2^, as higher angular velocities exceed the maximum bipolar frequency of the stepper motor. An advantage of CAP systems is that they allow for the creation of intricate flow patterns. As such, in the MoCAP device, we can also generate PSS profiles with minimum to maximum shear stress values ranging from 0.4 to 0.6, 0.7 to 1.3, 1.4 to 2.5, 2.8 to 5, 5.6 to 10, and 11.2–20 dyn/cm^2^ (Figure 2C). For experimental testing, we chose to focus on only shear stress profiles with a maximum shear stress value of 0.6 and 10 dyn/cm^2^ for CSS and PSS as these shear stress values approximate commonly used values on previous *in vitro* models of the BBB ranging from 0.4–12 dyn/cm^2^ (Meena et al., 2022; Santa-Maria et al., 2021; Suprewicz et al., 2022; Yeon et al., 2012; DeStefano et al., 2017; Peng et al., 2020; Harding et al., 2022). For PSS conditions, we chose to utilize an arbitrary frequency of 1 Hz (Hz) to mimic a normal adult resting heart rate (Nanchen, 2018), however, the frequency can be adjusted as needed for any system of interest.

**TABLE 2:** Angular velocities and corresponding maximum shear stress values.

MoCAP column position	Frequency (RPM)	Shear stress (dyn/cm^2^)
C1	28	0.6
C2	56	1.3
C3	112	2.5
C4	224	5
C5	448	10
C6	896	20

### 3.2 Impact of shear stress on BMEC passive barrier function

**FIGURE 3:**
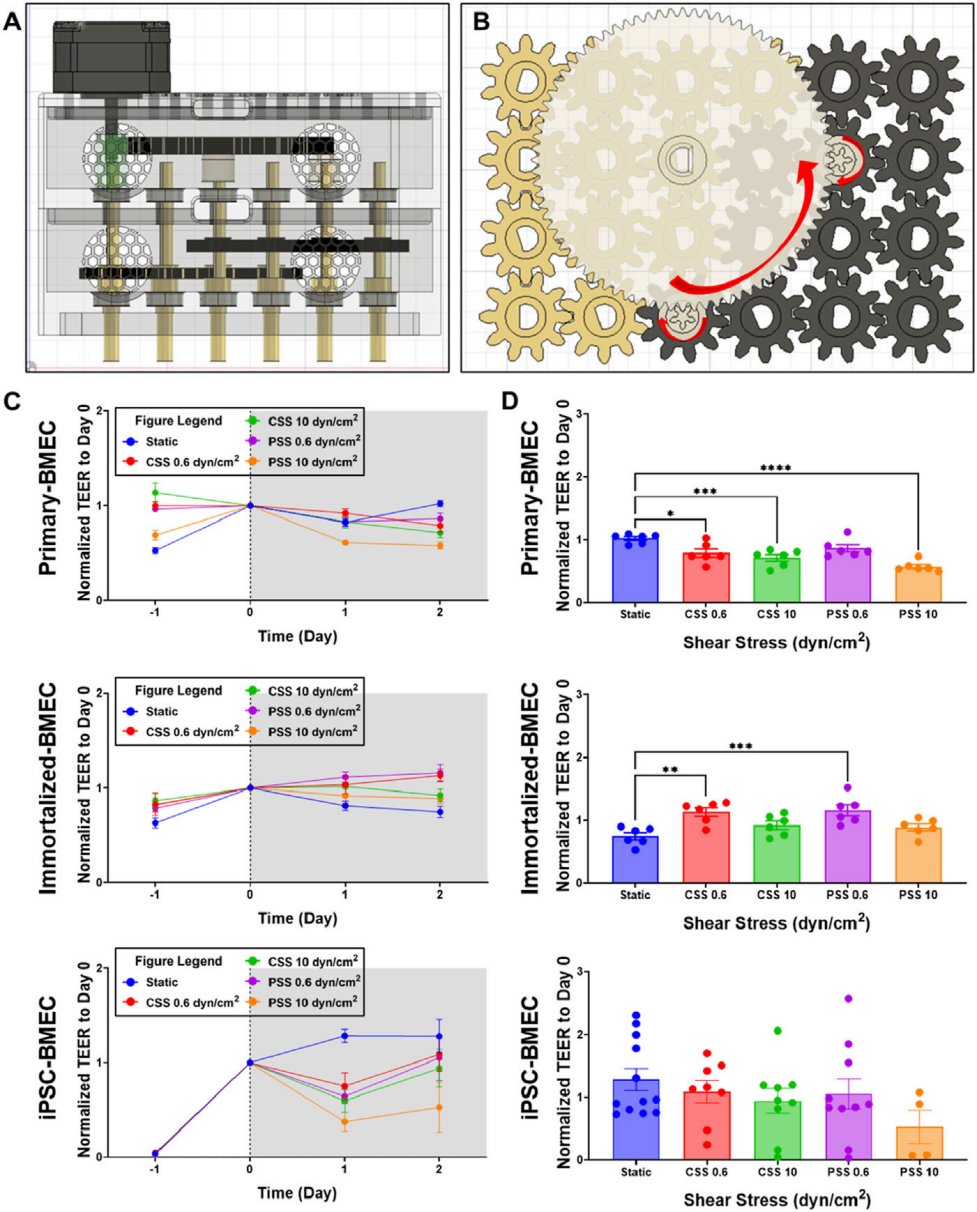
Effects of continuous and pulsatile shear stress on passive barrier function in BMECs. **(A, B)** Side view diagram **(A)** and top view of gear configuration **(B)** of MoCAP device configuration for applying 0.6 and 10 dyn/cm^2^ shear stress. **(C)** Daily TEER measurements of primary, immortalized, and iPSC-derived BMECs during the 2 days of continuous or pulsatile shear stress acclimation. For reference, cells are seeded on day −2 and shear stress is initiated on day 0. Data represent mean ± SEM from N = 6 (immortalized and primary-BMECs) and N = 4–12 (iPSC-derived BMECs) Transwell^®^ filters per condition. Any filter where cell detachment was observed at the end of the experiment was excluded from the analysis. **(D)** TEER summary for primary, immortalized, and iPSC-derived BMECs after 2 days of exposure to shear stress. Data represent mean ± SEM from N = 4–12 Transwell^®^ filters per condition, aggregated across two independent MoCAP runs. Statistical significance was calculated using a one-way ANOVA applied to each cell type (*, p < 0.05; **, p < 0.01; ***, p < 0.001; ****, p < 0.0001).

To demonstrate the capabilities of the MoCAP, we conducted exploratory evaluations into the effects of CSS and PSS on BBB function across several common BMEC sources (primary, immortalized, and iPSC-derived). The MoCAP device was reconfigured with a 1 to 16 gear reduction ratio (Figure 3A). In this configuration, the motor drives the gold gears at an angular velocity of 28 RPM creating 0.6 dyn/cm^2^ (Figure 3B). The white gears inside the top compartment of the MoCAP device then increase the input angular velocity and transfer it down to the black gears increasing the angular velocity to 448 RPM and thus generating 10 dyn/cm^2^ of shear stress inside the Transwell^®^ inserts (Figure 3B). In this configuration, the application of shear stress to the cell culture monolayer is almost instantaneous, with a small millisecond delay (13 m) caused by the gear backlash within the MoCAP gearbox (Supplementary Figure S3). We exposed cells to CSS and PSS for 2 days, utilizing a high and low threshold described above (10 and 0.6 dyn/cm^2^, respectively). We included dextran at a concentration of 6.5% (w/w), which increased media viscosity to 3 mPa based on rheology data from previous studies (Rouleau et al., 2010; Li et al., 2008). This allowed us to run the MoCAP device at a lower angular velocity to minimize the incorporation of bubbles into the cell media, while still achieving our desired shear stresses. Daily TEER measurements were collected throughout the course of the experiment for each cell line (Figure 3C). After 2 days of CSS and PSS acclimation, final TEER measurements were collected and compared across shear stress conditions within the same cell line. For the primary BMECs, there was a statistically significant decrease in TEER in the 0.6 dyn/cm^2^ CSS, 10 dyn/cm^2^ CSS, and 10 dyn/cm^2^ PSS conditions when compared to the static control group (Figure 3D). There was a statistically significant increase in TEER for the immortalized BMECs exposed to 0.6 dyn/cm^2^ CSS and 0.6 dyn/cm^2^ PSS (Figure 3D). It is important to note that the magnitude of TEER differences in the primary and immortalized BMECs was very low, since these cells have poor passive barrier properties (Supplementary Figure S4). In contrast, for the iPSC-BMECs, which have comparably higher baseline TEER (Supplementary Figure S4), there were no statistically significant differences detected after 2 days of CSS or PSS acclimation. Upon visual inspection, approximately 65% of the filters had intact monolayers regardless of exposure to PSS or CSS, and we anticipate this number could be improved with additional optimization of media and culture conditions. Only filters with intact monolayers were utilized for downstream cellular and molecular analyses.

### 3.3 Impact of shear stress on BMEC nuclei density and morphology

**FIGURE 4:**
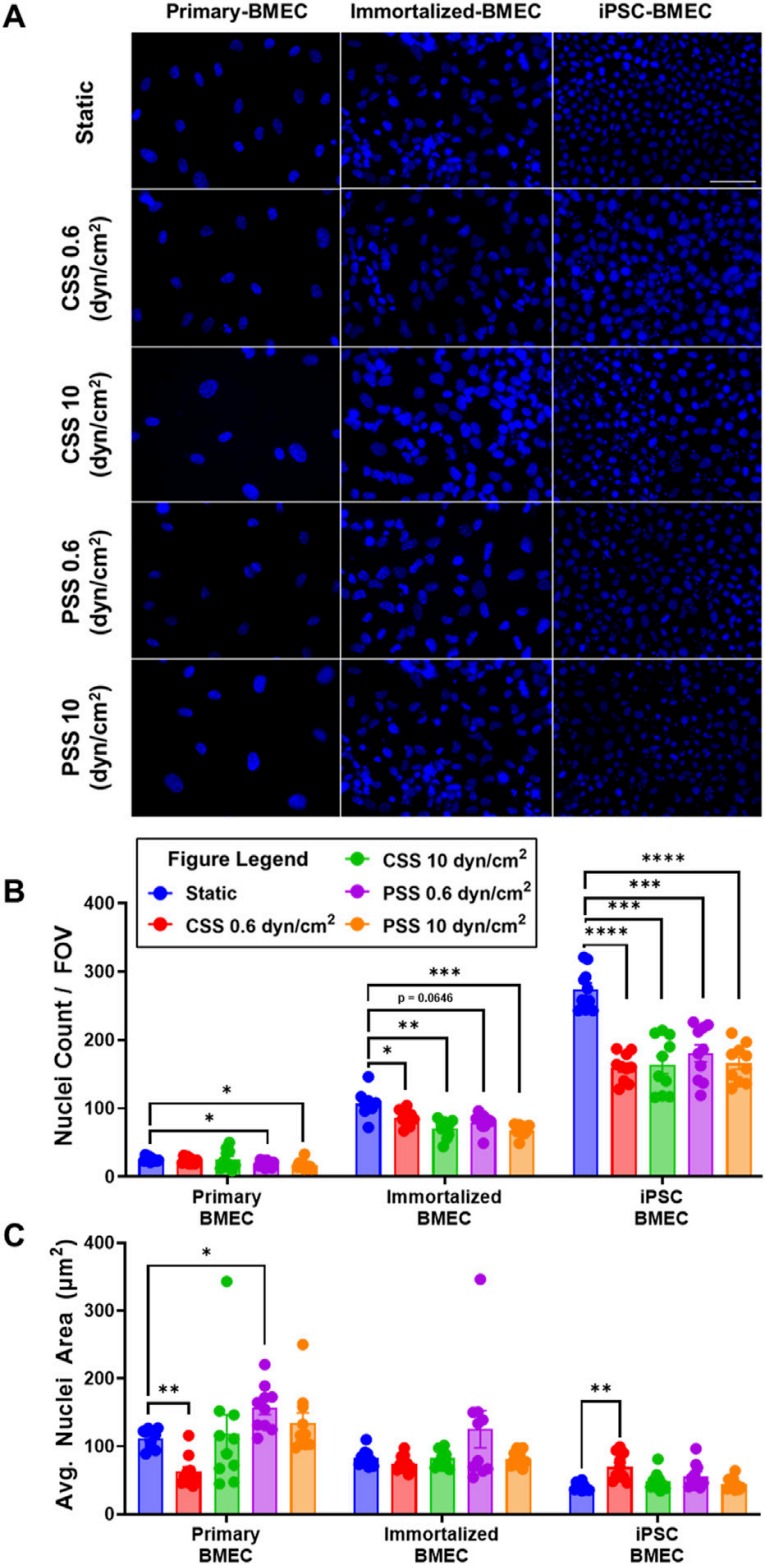
Effects of pulsatile and continuous shear stress on nuclei morphology in BMECs. **(A)** Nuclei visualization with DAPI in primary, immortalized, and iPSC-derived BMECs after 2 days of shear stress treatment. Scale bar indicates 100 µm. **(B)** Quantification of number of cell nuclei per field of view. **(C)** Quantification of average cell nuclei area. In **(B, C)**, data represent mean ± SEM from N = 10 Transwell^®^ filters per condition, aggregated across two independent MoCAP runs. Statistical significance was calculated using a one-way ANOVA applied to each cell type (*, p < 0.05; **, p < 0.01; ***, p < 0.001; ****, p < 0.0001).

To determine whether CSS and PSS exposure affected nuclear morphology, we analyzed the three BMEC lines using a DAPI nuclear stain (Figure 4A). The number of cell nuclei per field of view (FOV) was counted and analyzed, which first revealed statistically significant differences in cell nuclei numbers between all cell lines (Supplementary Figure S5A), indicative of different packing densities. In terms of responsiveness to shear stress, we observed a statistically significant decrease in the number of cell nuclei per FOV for the primary BMECs acclimated to 0.6 dyn/cm^2^ PSS, and 10 dyn/cm^2^ PSS when compared to the static primary BMEC control (Figure 4B). The analysis also revealed a statistically significant decrease in cell nuclei count per FOV in all the immortalized BMECs acclimated to shear stress when compared to their corresponding static control (Figure 4B). Further, there was a statistically significant decrease in cell nuclei per FOV in all iPSC-BMECs acclimated to shear stress when compared to their respective static control (Figure 4B). These results suggest a change in cell density induced by different shear stress conditions within all the cell lines. We next analyzed the average nuclei area. Here, we observed a statistically significant decrease in cell nuclei area for primary BMECs acclimated to 0.6 dyn/cm^2^ CSS when compared to the statically cultured primary BMECs (Figure 4C). There was also a statistically significant increase in cell nuclei area for primary BMECs acclimated to 0.6 dyn/cm^2^ PSS and iPSC-derived BMECs acclimated to 0.6 dyn/cm^2^ CSS when compared to their respective static control (Figure 4C). Cell nuclei area remained constant for all other shear stress conditions in all three BMEC lines when compared to their respective static control groups (Figure 4C), although we further note a statistically significant difference between the statically cultured primary, immortalized, and iPSC-BMEC average cell nuclei area (Supplementary Figure S5B), mirroring the differences in cell density between the lines. Overall, our results illustrate differential responses of the BMEC lines to shear stress, while all BMEC lines are resistant to shear stress induced nuclear shrinkage, which has been previously noted to occur in other cell types (Sahni et al., 2023; Jetta et al., 2019; Jin et al., 2020).

### 3.4 Impact of shear stress on GLUT1 expression

**FIGURE 5:**
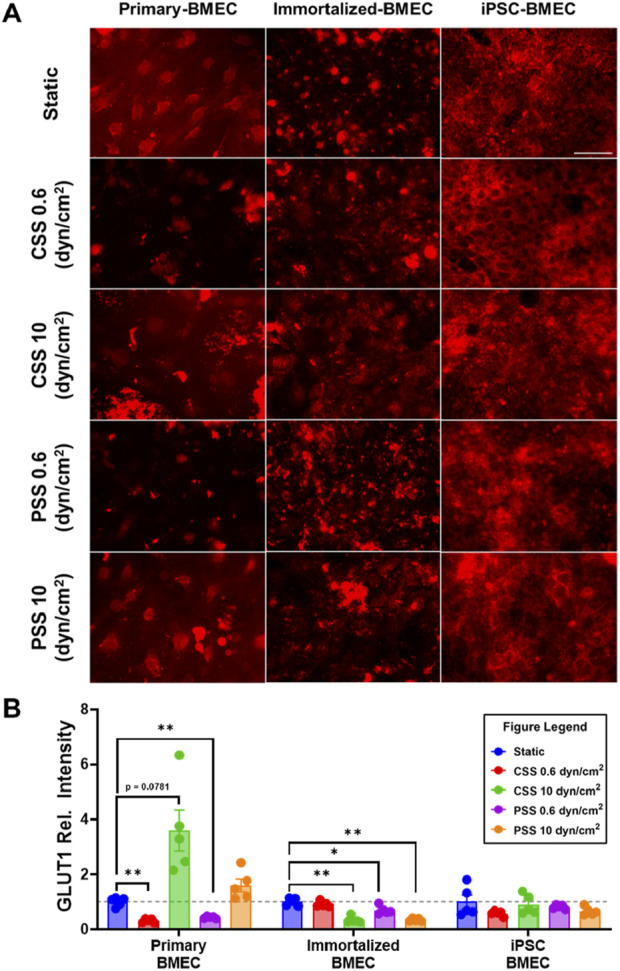
Effects of pulsatile and continuous shear stress on GLUT1 expression in BMECs. **(A)** Immunofluorescent staining of GLUT1 in primary, immortalized, and iPSC-derived BMECs after 2 days of shear stress treatment. Scale bar indicates 100 µm. **(B)** Quantification of GLUT1 expression. For each condition, fluorescence intensity from each treated group was normalized to the static control within a given cell line (grey dotted line). Data represent mean ± SEM from N = 5 Transwell^®^ filters per condition from a single MoCAP run. Statistical significance was calculated using a one-way ANOVA applied to each cell type (*, p < 0.05; **, p < 0.01).

We investigated the effects of CSS and PSS on glucose transporter 1 (GLUT1) expression, as previous reports noted an upregulation in GLUT1 transporter expression when primary BMECs were exposed to 10 dyn/cm^2^ of CSS, (Chavarria et al., 2023; Garcia-Polite et al., 2017), whereas GLUT1 expression in iPSC-BMECs is reported to be insensitive to CSS (DeStefano et al., 2017). We repeated the previously mentioned shear stress experiment and then performed immunofluorescent staining for GLUT1 (Figure 5A). The quantification of pixel intensity of GLUT1 showed a statistically significant decrease in primary BMECs exposed to 0.6 dyn/cm^2^ CSS and 0.6 dyn/cm^2^ PSS compared to their respective static control, as well as an increase in response to 10 dyn/cm^2^ CSS that was not quite statistically significant (p = 0.0781) (Figure 5B). There was also a statistically significant decrease in GLUT-1 intensity in the immortalized BMECs exposed to 10 dyn/cm^2^ CSS, 0.6 dyn/cm^2^ PSS, and 10 dyn/cm^2^ PSS compared to their respective static control (Figure 5B). No significant differences were noted in the iPSC-BMECs between any of the experimental conditions. Thus, our results are generally consistent with the published literature.

### 3.5 Impact of shear stress on BMEC morphology and alignment

**FIGURE 6:**
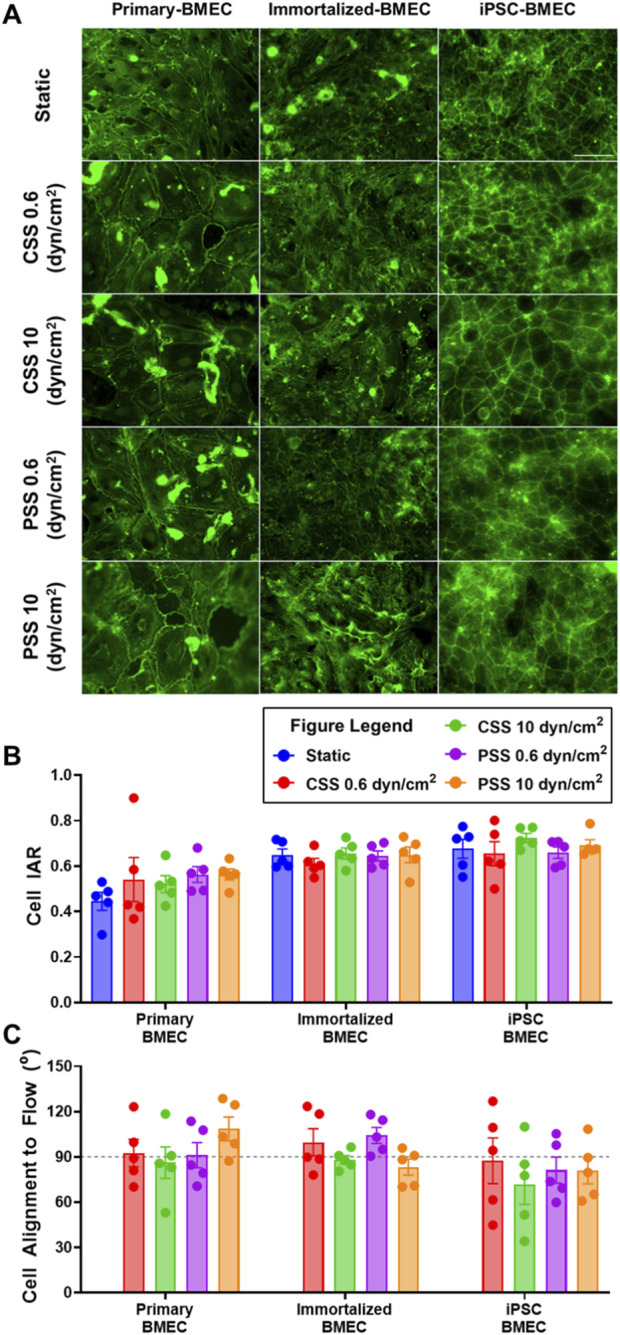
Effects of pulsatile and continuous shear stress on actin alignment in BMECs. **(A)** Images of actin cytoskeleton in primary, immortalized, and iPSC-derived BMECs after 2 days of shear stress treatment. Scale bar indicates 100 µm. **(B)** Quantification of cell inverse aspect ratio (IAR). **(C)** Quantification of cell alignment angle relative to flow direction, where the grey line represents cell alignment perpendicular (90°) to the fluid flow vector generated by the MoCAP device. In **(B, C)**, data represent mean ± SEM from N = 5 Transwell^®^ filters per condition from a single MoCAP run. Statistical significance was calculated using a one-way ANOVA applied to each cell type (no differences).

To examine the impact of shear stress on BMEC cytoskeleton morphology, we additionally performed immunofluorescent staining of the actin cytoskeleton utilizing a fluorescently labeled phalloidin after the cells had been exposed to CSS or PSS (Figure 6A). From the actin labeling, we manually measured cell width and length to calculate the inverse aspect ratio (IAR) and evaluate morphological changes in each BMEC line exposed to CSS or PSS. The IAR of each BMEC line remained constant irrespective of the shear stress condition it was exposed to (Figure 6B). The primary BMECs cultured under static conditions had significantly smaller IAR compared to immortalized BMECs and iPSC-BMECs (Supplementary Figure S6). This finding implies baseline differences in IAR between cell lines under static conditions. We then proceeded to calculate cell orientation based on the angle created between the flow vector and the length measured of each cell. This cell orientation measurement was not calculated on the static control groups as these cells were not exposed to flow conditions and did not have a flow vector. This analysis revealed that all cell lines, regardless of shear stress condition, align approximately perpendicular to the flow direction (∼90°; Figure 6C).

## 4 Discussion

In this study, we have developed a modular, versatile, and cost-effective mechanofluidic device that is compatible with commercial 24-well Transwell^®^ inserts. The device can introduce continuous and pulsatile shear stress conditions in a Transwell^®^ insert across a range of shear stress magnitudes. Since the MoCAP is compatible with Transwell^®^ inserts, this device allows for the exploration of the effects of shear stress on barrier-forming cells. The MoCAP can therefore aid mechanofluidic studies by rapidly testing shear stress conditions in 2D in conjunction with molecular biology assays before moving into more intricate 3D models such as microfluidics.

To demonstrate the utility of the MoCAP system, we conducted exploratory evaluations of the responses of three BMEC lines (primary, immortalized, and iPSC-derived) to different shear stress profiles. Similar to prior reports, we report shear-induced changes to GLUT1 expression in primary and immortalized BMECs, but not iPSC-BMECs (Chavarria et al., 2023; Garcia-Polite et al., 2017). These results illustrate the importance of considering the differences in cell line responses to shear stress in mechanobiological experiments. Further, all three BMEC lines aligned perpendicular to the flow direction in our cone-and-plate device acclimated on Transwell^®^ inserts, which agrees with a previous report on immortalized BMECs (Choublier et al., 2022). However, there are also contradictory reports on cell alignment with respect to flow direction for immortalized BMECs (Choublier et al., 2021), primary BMECs (Garcia-Polite et al., 2017) and iPSC-BMECs (DeStefano et al., 2017; Motallebnejad et al., 2019; Reinitz et al., 2015). These differences may be due to the mechanofluidic devices utilized during testing, which apply shear forces in different ways. We also found that all three BMEC lines did not experience any elongation due to flow as seen by the IAR measurements when compared to their respective static control, which is consisted with literature (DeStefano et al., 2017; Reinitz et al., 2015; Bogorad et al., 2017). In addition, all BMEC lines exhibit decreased cell density in response to shear stress, which could be related to cell packing or altered proliferation, but more experiments will be needed to tease out these effects. Lastly, we found that the immortalized BMECs had a subtle but significant increase in TEER measurements when exposed to low shear stress, which is consistent with previously reported results (Kim et al., 2024). Overall, these experiments highlight that the MoCAP enables higher throughput evaluation of different cellular and molecular properties after exposure to a range of shear stresses.

Although we have only presented a limited number of molecular assays, the MoCAP can enable the incorporation of shear stress in molecular assays that have been traditionally performed under static conditions. For example, shear stress plays an important role in cancer metastasis (Qin et al., 2021; Dombroski et al., 2021; Kim O.-H. et al., 2022; Zhou et al., 2023; Huang et al., 2018; Bouchalova and Bouchal, 2022; Spencer and Baker, 2016; Spencer et al., 2021), therefore traditional migration and invasions assays performed in Transwell^®^ inserts can now be performed with external shear stress by utilizing the MoCAP device. This would enable scientists to screen the effects of different shear stress magnitudes and flow profiles on the metastatic potential of different cancer cell lines. Additionally, the MoCAP device can be used with other cell lines from different tissues in which the incorporation of flow is important for cellular function. Some potential examples include the blood-cerebrospinal fluid barrier (MacAulay et al., 2022; Solár et al., 2020), liver (Sun et al., 2019; Poisson et al., 2017; Duan et al., 2022), and peripheral vascular system (Obi et al., 2014; He et al., 2022; Chistiakov et al., 2017; Kutikhin et al., 2018; Ryu et al., 2021; Bertani et al., 2021; Voyvodic et al., 2014; Le et al., 2021; Le et al., 2021; Chatzizisis et al., 2011). Overall, we have presented a new mechanofluidic device that is cost effective, versatile, and incorporates shear stress into widely used Transwell^®^ models. We anticipate this tool will be broadly useful to the mechanobiology research community.

## Data Availability

The raw data supporting the conclusions of this article will be made available by the authors, without undue reservation.
