# Supplementary material for: Modular cone-and-plate device for mechanofluidic assays in Transwell inserts
Source: Front Bioeng Biotechnol. 2025 Jan 27;13:1494553. doi: 10.3389/fbioe.2025.1494553 (PMC11807968; doi:10.3389/fbioe.2025.1494553)
Supplement: Supplementary file 1 [file DataSheet1.docx]

# Supplementary Figures

^1,2^

**Supplementary Figure 1: Determining Cell Media Viscosity by Dextran Concentration.** The desired media viscosity was determined by collecting data from the literature^1,2^ performing a nonlinear regression, and calculating the desired dextran concentration.


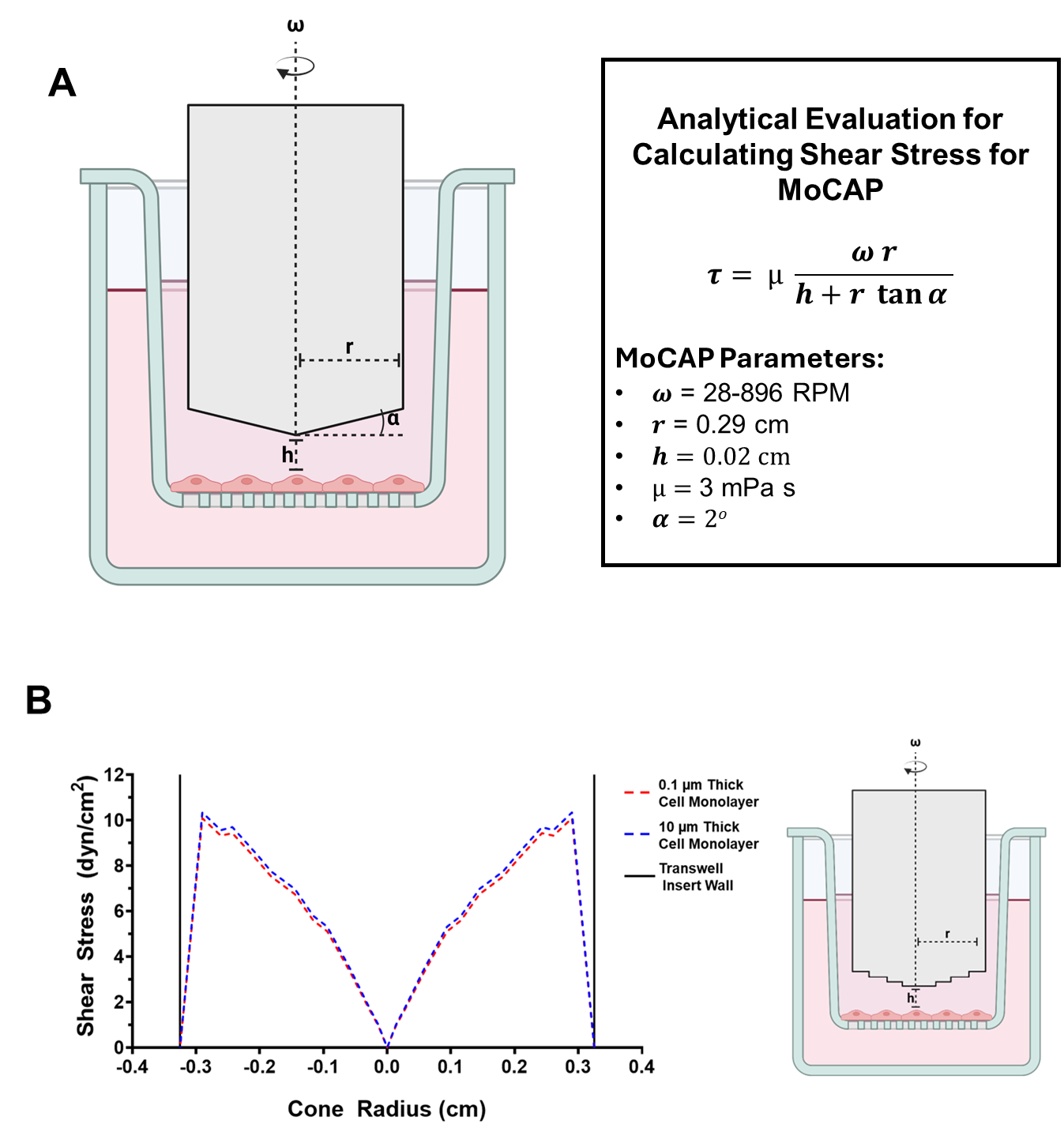


**Supplementary Figure 2: Analytical Evaluation of Shear Stress Created by MoCAP Device. (A)** Diagram of a single cone from the MoCAP device inserted into a Transwell insert explaining the experimental parameters to calculate the shear stress induced. Here, 𝜏 is shear stress, µ is the dynamic viscosity of the cell media, 𝜔 is angular velocity, ℎ is the gap between the tip of the low angle cone and the cells, 𝑟 is the cone radius, and 𝛼 is the angle of the cone. **(B)** Analytical evaluation of 3D printed low angles cones utilized in MoCAP device programmed to angular velocity of 448 RPM to create a maximum shear stress of 10 dyn/cm^2^, accounting for differences in cell monolayer thickness (0.1 µm and 10 µm). The jagged profile of the shear stress generated at the cell surface is due to the 3D printing resolution resulting in a cone with ascending steps instead of a smooth slope. Created in BioRender. Chavarria, D. (2025) https://BioRender.com/t53u482

**Supplementary Figure 3: Shear Stress Delay Created by Gear Backlash.** The gears within the system have a backlash of 2.2^o^, leading to a delay of 13.1 ms when the device generates 0.6 dyn/cm^2^ pulsatile shear stress and a delay of 13.2 ms when the device generates 10 dyn/cm^2^ of pulsatile shear stress.

**
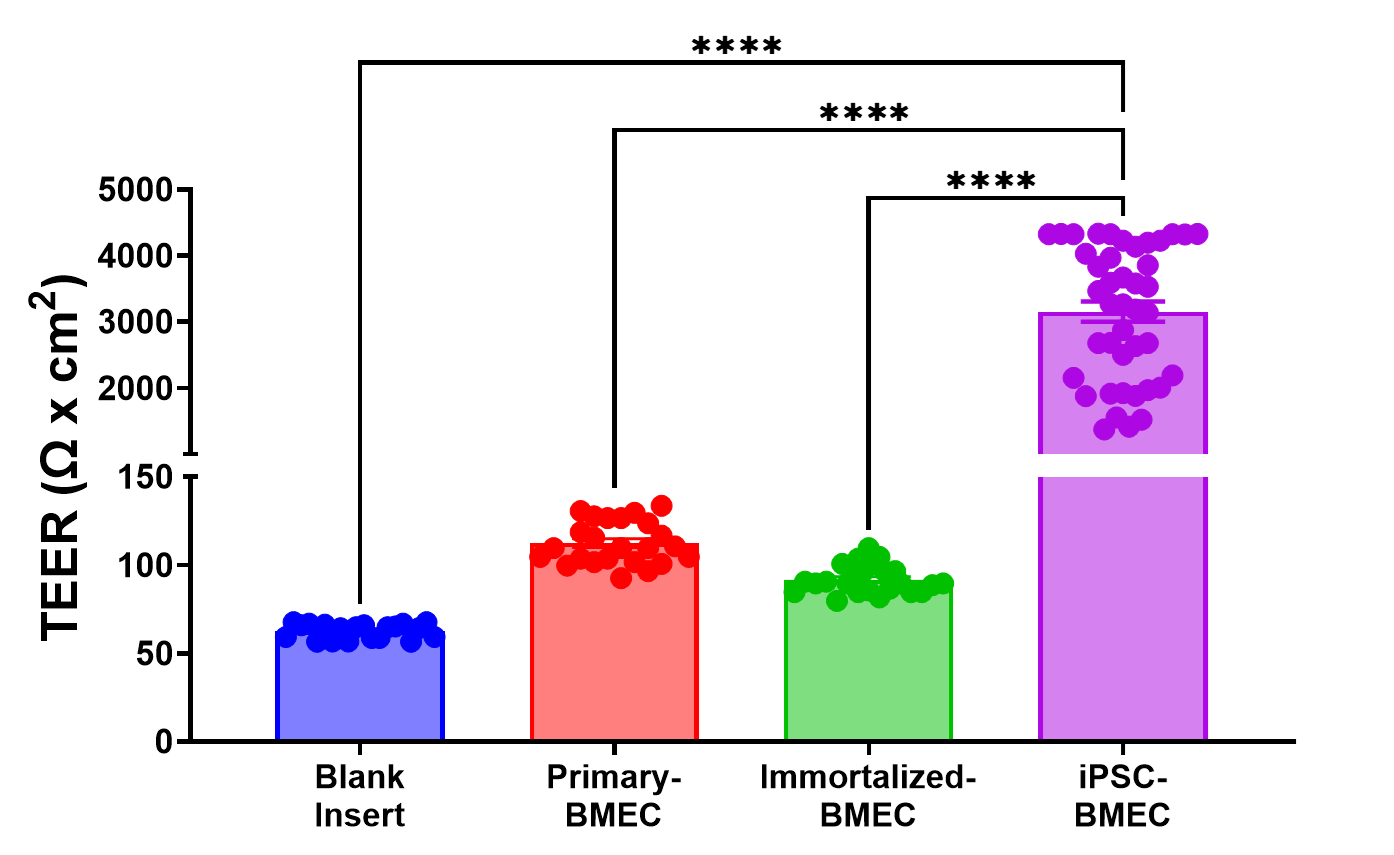
**

**Supplementary Figure 4: TEER Measurements of Different Cell Lines and Blank Transwell® Insert at Day 0.** TEER measurements of a control blanks Transwell® insert, Primary-BMEC, Immortalized-BMEC, and iPSC-derived BMECs at Day 0 before the application of different shear stress profiles using the MoCAP. Data represent mean ± SEM from N=20 for Blank Insert, N=24 for Primary and Immortalized-BMEC, and N=43 for iPSC-BMEC. Transwell filters per condition, aggregated across four independent MoCAP runs. Statistical significance was calculated using a one-way ANOVA (****, p<0.0001).


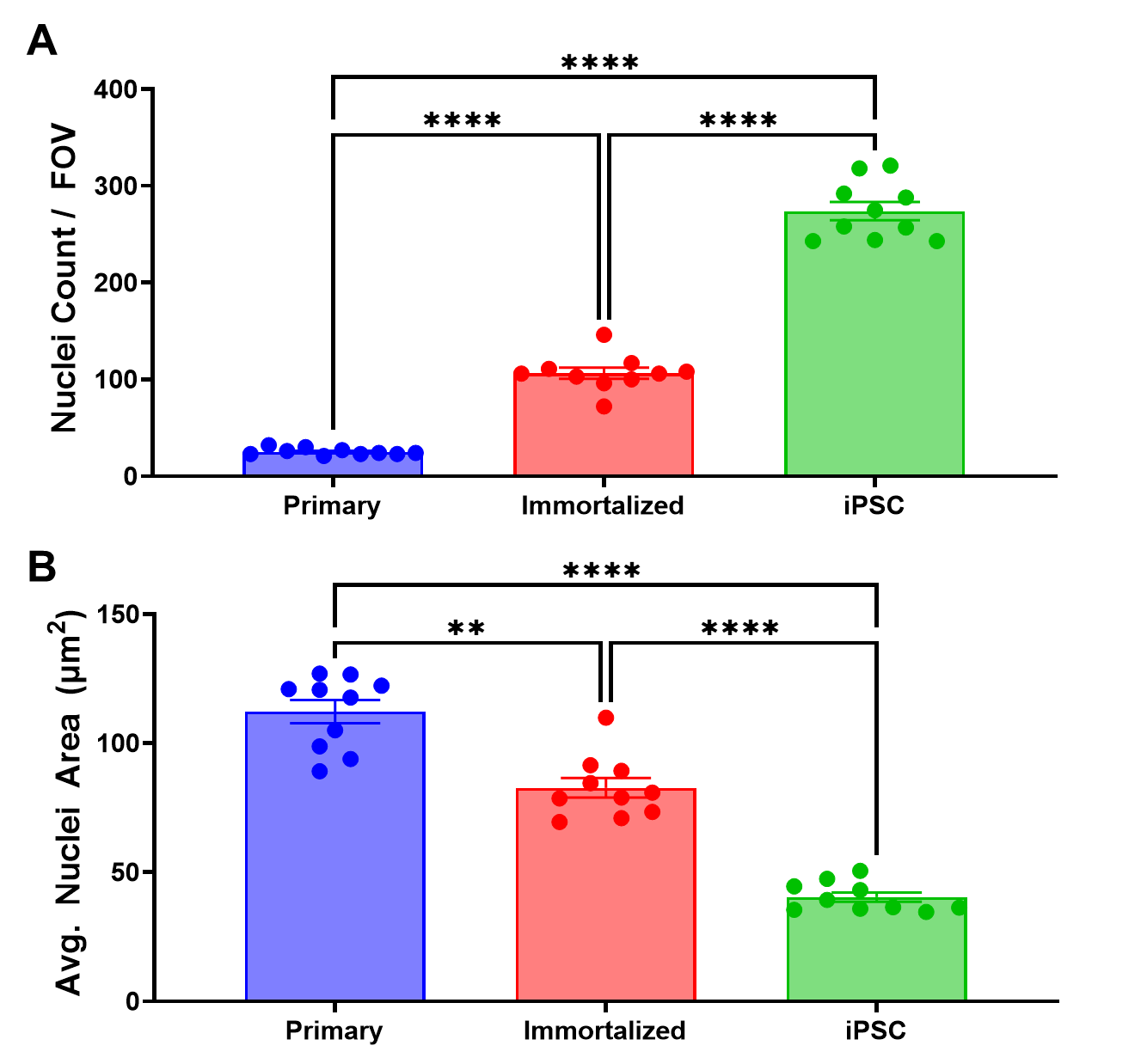


**Supplementary Figure 5: Differences in nuclear density and morphology between BMEC lines.** Quantification of cell nuclei count per field of view **(A)** and cell nuclei area **(B)** of statically cultured primary, immortalized and iPSC-derived BMECs. Data represent mean ± SEM from N=10 Transwell filters per condition, aggregated across two independent MoCAP runs. Statistical significance was calculated using a one-way ANOVA (**, p<0.01; ****, p<0.0001).


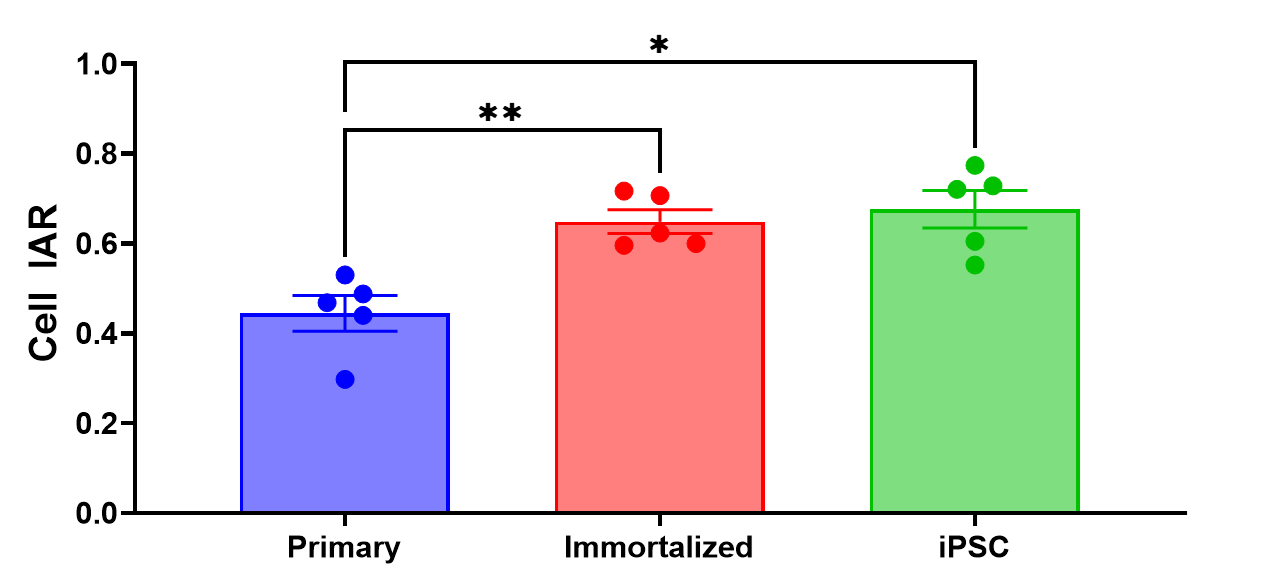


**Supplementary Figure 6. Differences of inverse aspect ratio between BMEC lines.** Quantification of the inverse aspect ratio of statically cultured primary, immortalized, and iPSC-derived BMECs. Data represent mean ± SEM from N=5 Transwell filters per condition from a single MoCAP run. Statistical significance was calculated using a one-way ANOVA (*, p<0.05; **, p<0.01).

**References**

1. Rouleau, L., Rossi, J. & Leask, R. L. Concentration and Time Effects of Dextran Exposure on Endothelial Cell Viability, Attachment, and Inflammatory Marker Expression In Vitro. *Ann Biomed Eng* **38**, 1451–1462 (2010).

2. Li, D., Dai, K. & Tang, T. Effects of dextran on proliferation and osteogenic differentiation of human bone marrow-derived mesenchymal stromal cells. *Cytotherapy* **10**, 587–596 (2008).
